# Field evaluation of entomopathogenic fungi and Usher plant *Calotropis procera* extract for controlling aphids and whiteflies on pepper

**DOI:** 10.1038/s41598-025-06100-y

**Published:** 2025-07-05

**Authors:** Samy M. Sayed, Monir M. El Husseini, Essam Agamy, Marwa M. A. Farag

**Affiliations:** Center of Biological Control & IPM, Department of Economic Entomology and Pesticides, Faculty of Agriculture, Cairo University, Giza, 12613 Egypt

**Keywords:** *Calotropis procera*, *Beauveria Bassiana*, *Metarhizium anisopliae*, *Bemesia tabaci*, *Myzus persicae*, Biological techniques, Ecology

## Abstract

Both plant extracts and entomopathogenic fungi play a crucial role in sustainable agriculture as environmentally friendly alternatives to synthetic pesticides. The study aimed to use *Calotropis procera* extract (PE), and the commercial entomopathogenic fungi; *Beauveria bassiana* (*Bb*) and *Metarhizium anisopliae* (*Ma*) individually and each one with PE against both aphid and whitefly on pepper plants in two locations. Phenols and flavonoids in leaves of *C. procera* were detected by HPLC analysis and indicated that there are 14 compounds and some of them are showing insecticide effects. This extract showed LC_50_ and LC_90_ on *Myzus persicae* as 292.24 and 709.7 µg/mL, respectively. Field experiments showed that reduction rates for aphids were increased with significant differences among the treatments in the location-1 to be 100% (PE + *Bb*), 97.47% (PE + *Ma*) and 95.53% (pesticide), 87.83% (*Bb*), and 81.29% (PE) followed by lower rate of 75.98% (Ma). These reduction rates in the location (2) were 96.1% (PE + *Bb*), and 93.4% (pesticide). Meanwhile, the reduction rate of PE + *Ma* (86.4%) was significantly lower than that of PE + *Bb* and pesticide but higher than that of PE (78.23%). Similar trend was recorded) for whitefly in both locations where it was 96% for PE + *Bb* and pesticide followed by *Bb*, PE and PE + *Ma* (86–88%) while the lower rate was 77.48% (Ma). Meanwhile, the reduction rates in the location-2 reached 100% for PE + *Bb*, PE + *Ma* and pesticide with significant differences for *Bb* (96.11%) followed by the lower two treatments (PE = 87.98% and Ma = 89.76%) without a significant difference between both of them. It could be concluded that the using of the commercial EPF especially *B. bassiana* with *C. procera* leaf extract achieved the higher reduction rates of aphids and whiteflies than that of using of each one individually. Accordingly, they could be used together in integrated pest management of piercing sucking insect pests due to there are synergistic effects and compatibilities between both of them. Moreover, other isolates of EPFs should be tested with this plant extract to ensure from the compatibility between them due to some EPFs isolates are not compatible with some plant extracts.

## Introduction

Aphids and whiteflies are common pests that can severely damage pepper plants (*Capsicum* spp.). Both pests feed on sap of the plant causing stunted growth, yellowing of leaves, and potential plant death if left unprotected. They can also transmit plant viruses, further exacerbating the damage. They also reduce plant growth, lower yields, and decrease the marketability of vegetables that causes economic losses for farmers^1,2^.

Plant extracts are increasingly being used in Integrated Pest Management (IPM) strategies as an environmentaly safe and sustainable approach to pest control^3^. IPM focuses on using a combination of biological, cultural, mechanical, and chemical control methods, with an emphasis on minimizing the use of chemical pesticides to reduce environmental and health risks^4^. Many plant extracts contain compounds that can act as natural insecticides. For example, compounds like pyrethrins and neem oil are well-known for their insecticidal properties^5^.

As antifungal and antibacterial properties, plant extracts proved to control plant pests and diseases without the need for synthetic chemicals^6^. Some plant extracts can act as natural repellents to keep pests away from crops, Such as Citronella oil and Peppermint oil^7^. These extracts can be used to target specific pests while being less harmful to beneficial insects and the environment. One of the major benefits of using plant extracts in IPM is that pests are not developing resistance to them compared to synthetic chemical pesticides. The compounds in plant extracts often target multiple pathways or behaviors in pests, making it harder for pests to adapt and build resistance. This makes them an attractive option for sustainable agriculture^8^.

*Calotropis procera* (also known as the Swan plant, Apple of Sodom, or Aak) produces toxic compounds, particularly cardenolides, in its sap, which can act as a natural pesticide. These toxins can deter herbivores of certain pests^9^. The extracts of this plant has been explored for its potential as a bio-pesticide. The toxic compounds in *C. procera* can be extracted and formulated into plant-based pest control products that can be used in organic farming or as an alternative to synthetic chemicals^10^.

Entomopathogenic fungi (EPF) acting as natural pathogens to insects, playing a crucial role in IPM systems due to thier ability to infect insects and penetrate the cuticle by producing enzymes that break down the insect’s exoskeleton. The fungus then grows inside the insect, prolefirating specific mycotoxins killing their hosts^11,12^. The valuable role of EPFs in IPM targeting specific pests without harming beneficial organisms maintaining the ecosystem health, while managing pest populations sustainably. With careful application and consideration of environmental factors, EPFs are an essential component of modern, sustainable agriculture. *Beauveria bassiana* and *Metarhizium anisopliae* are used as biological control agents to manage pest populations in an environmentally sustainable manner. These EPFs infects a wide variety of insect pests, including aphids, whiteflies, thrips, and caterpillars^13^.

The synergism between plant extracts and EPF in IPM is an exciting area of research and practice. Combining plant extracts with EPF can lead to a higher level of pest control, as both the chemical action of the plant extract and the biological action of the fungi are working together, and EPF may provide longer-lasting control since the fungus continues to spread and infect pests over time, while plant extracts provide an initial knockdown effect that enhance pest control effectiveness and promote a more sustainable approach to pest management^14,15^. Therefore, the current study aimed to evaluate *C. procera* leaf extract (PE), and two commercial EPF; *B. bassiana* (*Bb*) and *M. anisopliae* (*Ma*) individually and each with PE against aphid and whitefly on pepper plants in two locations at Giza Governorate, Egypt.

## Materials and methods

### Collection of Usher leaves and extraction

The green usher plant leaves (*C. procera*) were collected from the plants located at Giza (29° 36′ 58.16′′ N and 31° 19′ 53.76′′ E). Then, they were left for 10 days in the dark until they dried and ground to fine powder. Thereafter, 20 g of the the obtained powder were extracted with 400 ml absolute ethanol for 2 days at 35 °C. Then, the solution was cooled and centrifuged at 7000 rpm for 15 min. The solution was filtered 3 times with filter paper of Whatman No. 1 for collecting pure supernatant^16^. The pellet was dissolved in aqueous solution of DMSO (1%) with a final concentration of 1 g/L). The extract was stored at 4 °C until it used for the HPLC analysis, bioassay experiment and field application.

### HPLC analysis

To analyse and detect phenols and flavonoid compounds in leaves of *C. procera*, the procedure of Lu et al. was used^17^. Agilent 1260 infinity HPLC Series (Agilent, USA) was used and maintained with a quaternary pump. The column of a Kinetex® 5 μm EVO C18 100 mm × 4.6 mm, (Phenomenex, USA) was used with operation at 30 °C. A ternary linear elution gradient was used for the separation with (A) HPLC grade water (0.2%) and H_3_PO_4_ (v/v), (B) acetonitrile and (C) methanol. A volume of 20 µL from the extract was injected. In order to detect the flavonoids and phenols, AVWD detector set was used at 284 nm.

### Bioassay of plant extract on aphids

The extract of *C. procera* leaves was diluted to obtain high range of five concentrations of 125, 250, 500, 1000, 2000 µg/mL in order to obtain accurate LC_50_ and LC_90_. Aqueous DMSO (1%) was used in the control as a solvent and also it was added to all concentrations of the plant extract with the same ratio. Five ml of each concentration was sprayed using a fine sprayer with equal homogeneity on an area of 50 cm^2^. This area contained five leaves of pepper plant where 10 aphids were deposited on each leaf. Then, each leaf was placed in a Petri dish of 10 × 1.5 cm^18^. The individuals of the tested aphid, *M. persicae* were collected on the same day of the bioassay experiments from pepper plants infested with the aphids. Moistened cotton tissues were added in Petri dishes to maintain the humidity. Then, all dishes were kept at 26 ± 1 C°, 70 ± 5 RH and a photoperiod of14:10 L: D. Aphid mortality was recorded after 24 h.

### Entomopathogenic fungi

The commercial entomopathogenic fungi (EPF), i.e., Bio Power (*Beauveria bassiana* @ 1 × 10^9^ CFU’s/ gm) and Bio-Magic (*Metarhizium anisopliae* @ 1 × 10^9^ CFU’s/ gm) were used in the experiments. The recommended dose for application of vegetable crops is 6 gm/L water, therefore, 6 × 10^9^ CFU’s/ L water was applied. The two EPFs were assessed for the conidial viability where each product was diluted to 1 × 10^3^ conidia/ml. The dilution was carried as follow: one gram was dissolved in one litre, thereafter, one ml was disolved in 999 ml water, thus, the concentration of both fungi was 1 × 10^3^ conidia/ml. Then, 5 Petri dishes for each fungus were used containing 10 ml of culture medium (PDA). The fungal suspension (50 µl ) was distributed on the surface of each plate. After evaporation of the water, the plates were closed and incubated for germination and growth at 26 ± 1 °C under photophase of 14 h. After 72 h, the developed colonies were counted.

### Field application

The pepper varaiety (Mravel F1), was cultivated at two farms at Giza, Egypt at locations of (Location 1 at 29° 36’ 58.16’’ N and 31° 19’ 53.76’’ E, location 2 at 29° 34’ 11.69’’ N and 31° 19’ 48.26’’ E) as seedlings on September 02, 2024. On September 19, the experiments were started by six treatments carried out in each farm plus the control. In all treatments and the control, DMSO (1%) was added. Application of the tested agents occurred by weekly spraying. Treatment-1(PE) was *C. procera* extract with the concentration obtained from bioassay as LC90 (0.71 g/L); treatment-2 (*Ma*) with the recommended dose (6 gm/L water); treatment-3 (*Bb*) with the recommended dose (6 gm/L water); treatment-4 (PE *+ Ma*) with *M. anisopliae* (6 gm/L water) and *C. procera* extract (0.71 g/L); treatment-5 (PE + *Bb*) with *B. bassiana* (6 gm/L water) and *C. procera* extract (0.71 g/L) and treatment (6) as positive control by spraying every two weeks with the insecticide Delta 1 EC 5% at the recommended dose (80 ml/ 100 L water). In the treatments 4 and 5, the plant extract was sprayed at 8–9 a.m.; while the fungus was sprayed at 15–16 p.m. Each treatment and the control was in three plots each of 2 × 2 m in randomized complete block design (RCBD). All treatments were applied with the spray by BINDA sprayer (Taizhou Binda Plastic Co., Ltd., Taizhou, China). The aphid; *Myzus persicae* (Aphididae: Hemiptera) and whitefly; *Bemesia tabaci* (Aleyrodidae: Hemiptera) count was done weekly by randomized selection of three leaves (upper and lower surfaces) in three pepper plants in each plot (totally 9 leaves in each plot). In these two locations, the environmental conditions were as follow: Maximum temperature ranged from 27 to 40 and minimum temperature ranged from 17 to 27 °C. Humidity ranged from 60 to 68% while the day length ranged from 11 12 h. These conditions are suitable for fungi germination and pathogenecity.

### Statistical analysis

The mortality rates in bioassay were corrected using Abbott’s formula^19^ correlated with those of the control. The LC_50_ and LC_90_ values were obtained by Log-probit analysis of concentration versus mortality. Reductions were estimated with the formula of Henderson and Tilton^20^. Reduction rates were statistical analyzed by One-Way ANOVA and Duncan-test with α = 0.05. The statistical analysis was done using SPSS software program (version 23).

## Results

### Laboratory experiments

**Fig. 1:**
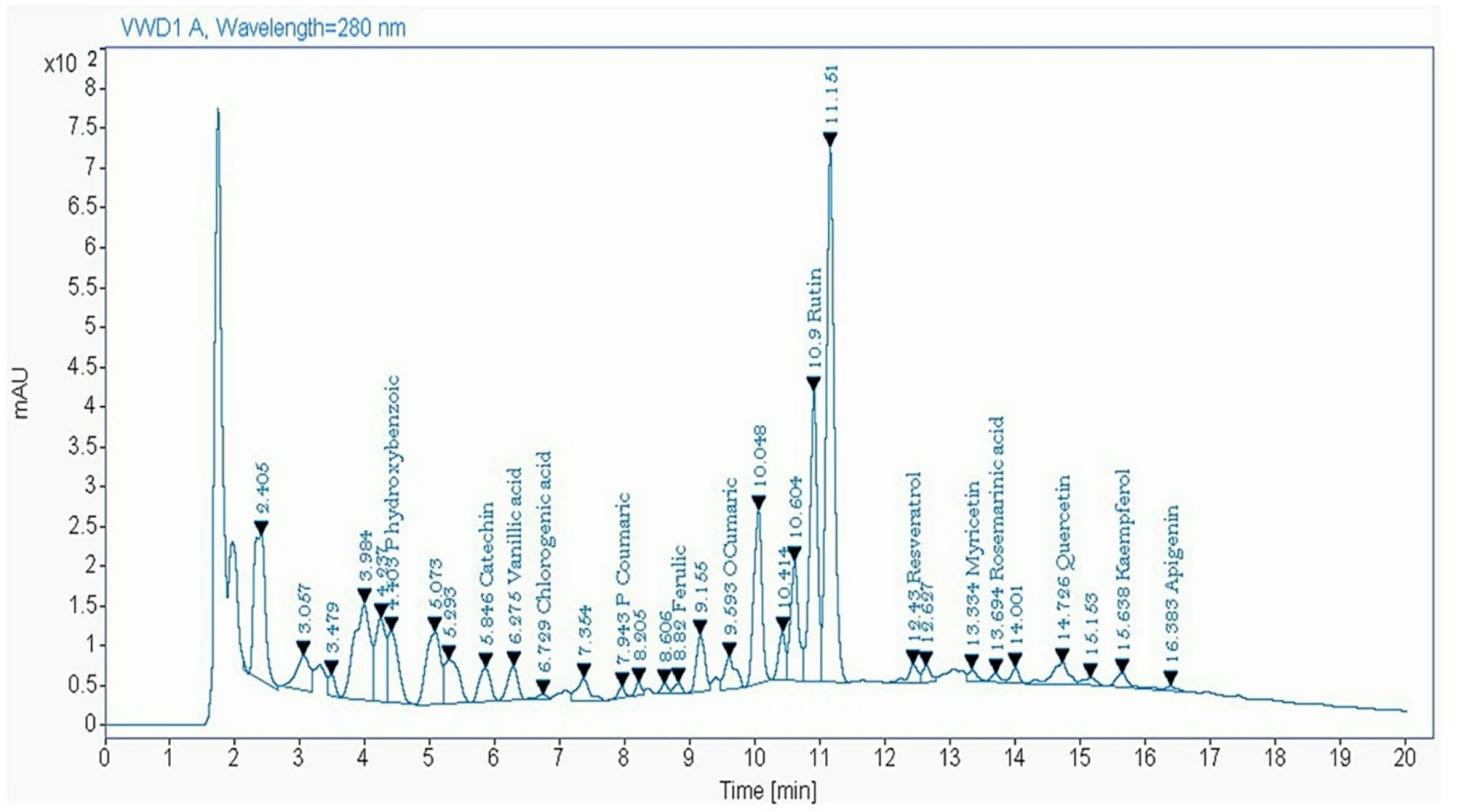
HPLC of ethanolic extract of *Calotropis procera* leaves.

Results of the HPLC analysis for *C. procera* ethanolic extract showed the presence of 14 components of phenols and flavonoids. The total phenols and flavonoids was 16.344 mg/g where Quercetin, Rutin, P- hydroxybenzoic and Catechin were the main components with 4.872, 3.85, 2.313 and 2.255 mg/g, respectively (Table 1; Fig. 1).

**Table 1:** Contents of phenols and flavonoids in ethanolic extracts of *Calotropis procera* leaves (mg/g).

Compounds	Retention time (Min)	Amount
P- hydroxybenzoic	4.4	2.313
Catechin	5.85	2.255
Vanillic acid	6.28	0.682
Chlorogenic acid	6.73	0.221
P Coumaric	7.94	0.06
Ferulic	8.82	0.33
O- Cumaric	9.59	0.257
Rutin	10.9	3.85
Resveratrol	12.43	0.603
Myricetin	13.33	0.13
Rosemarinic acid	13.69	0.355
Quercetin	14.73	4.872
Kaempferol	15.64	0.326
Apigenin	16.38	0.09
Total		16.344

The LC_50_ value of *C. procera* leaf extract on aphid adults of *M. persicae* was 292.24 µg/ml (CI = 246.9–334.9) while LC_90_ value was 709.7 µg/ml (CI = 635.9–813.6). The intercept = -0.89 ± 0.06, Slope = -15.22, χ^2^_(23)_ = 85.29, and *P* < 0.001. Accordingly, the LC_90_ as 0.71 g/l was used in the field experiments.

Regarding the conidial viability of the commercial EPF *B. bassiana*, it recorded 92 ± 0.89%, while it was 90.4 ± 1.41% for *M. anisopliae*.

### Field experiments

#### Aphids

**Fig. 2:**
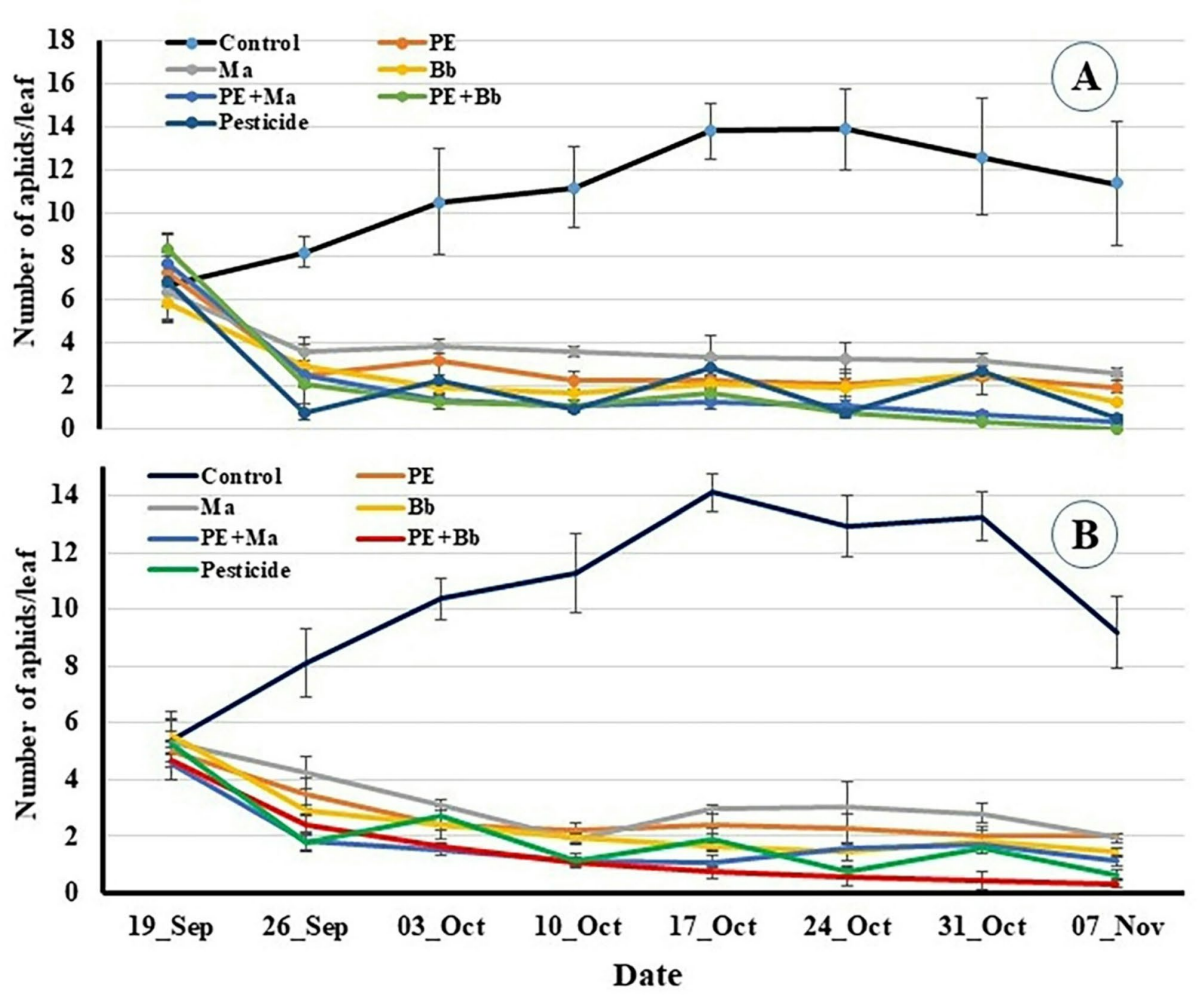
Population densities of aphid, *Myzus persicae* on pepper plants treated with entomopathogenic fungi and *Calotropis procera* extract at location-1(A) and location-2 (B).

Results showed that the infestation rates of aphids at the beginning of the experiment on control plants was 6.63 and 5.4 aphids/leaf for location 1 and 2, respectively. These infestation’s rates gradually increased till the 4th week and it reached 13.8 and 14.13 aphids/leaf, then, the infestation rate gradually decreased till the end of the experiment (11.37 and 9.2 aphids/leaf on November 07 for location 1 and 2, respectively) (Fig. 2A and B).

In treatments, aphid infestation in all treatments gradually decreased till end of the field study as follow: 1.91, 2.63, 1.27, 0.33, 0 and 0.53 aphids/ leaf while in location 2, they were 2, 1.93, 1.43, 1.13, 0.33 and 0.63 aphids/ leaf for PE, *Ma*, *Bb*, PE + *Ma*, PE + *Bb* and pesticide, respectively) (Fig. 2A and B).

**Fig. 3:**
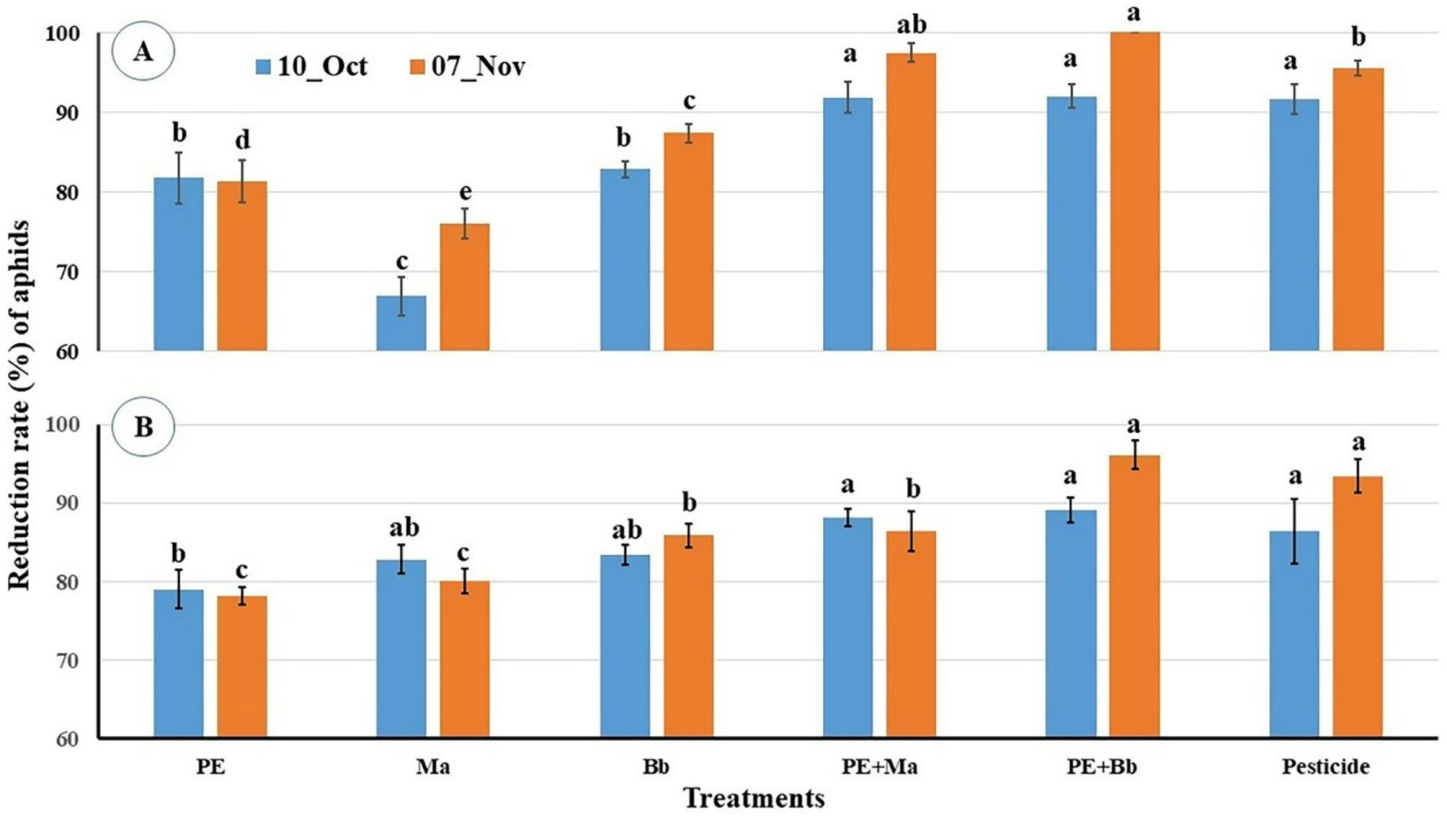
Reduction% in aphid numbers post treatment with entomopathogenic fungi and *Calotropis procera* extract. **A** = location-1; **B** = location-2. Columns of the same period bearing different letters are significantly different with Duncan test (*α* = 0.05).

Reduction rates after three weeks of treatments showed that there are significat differences among them in location-1 (F_5,12_ = 64.66, P < 0.001) where PE + *Ma*, PE + *Bb* and pesticide achieved reduction rates about 91.5 and they were significantly higher than that of *Bb* (82.84%) and PE (81.7%) follwed by the lower rate of *Ma* (66.87%) (Fig. 3A). At end of the experiment, reduction rates increased with significant differences among the treatments (F_5,12_ = 116.43, P < 0.001) to be 97.47% (PE + *Ma*), 100% (PE + *Bb*) and 95.53% (pesticide), 87.83% (*Bb*), and 81.29% (PE) follwed by a lower rate of 75.98% (*Ma*). In the location-2, the same trend was achieved where the reduction rates after three weeks of treatments showed significat differences among them (F_5,12_ = 3.89, *P* = 0.025), where PE + *Ma*, PE + *Bb* and pesticide achieved reduction rates about 86 to 89% and they were significantly higher than that of *Bb* (83.37%) and *Ma* (82.82%); while PE recorded the lower rate (78.22%). These reduction rates at the end of the experiment increased with significant differences among the treatments (F_5,12_ = 45.3, P < 0.001) to be 96.1% (PE + *Bb*), and 93.4% (pesticide), meanwhile PE + *Ma* (86.4%) was significantly lower than those of PE + *Bb* and pesticide. The lower rate was also for PE (78.23%) (Fig. 3B).

### Whiteflies

**Fig. 4:**
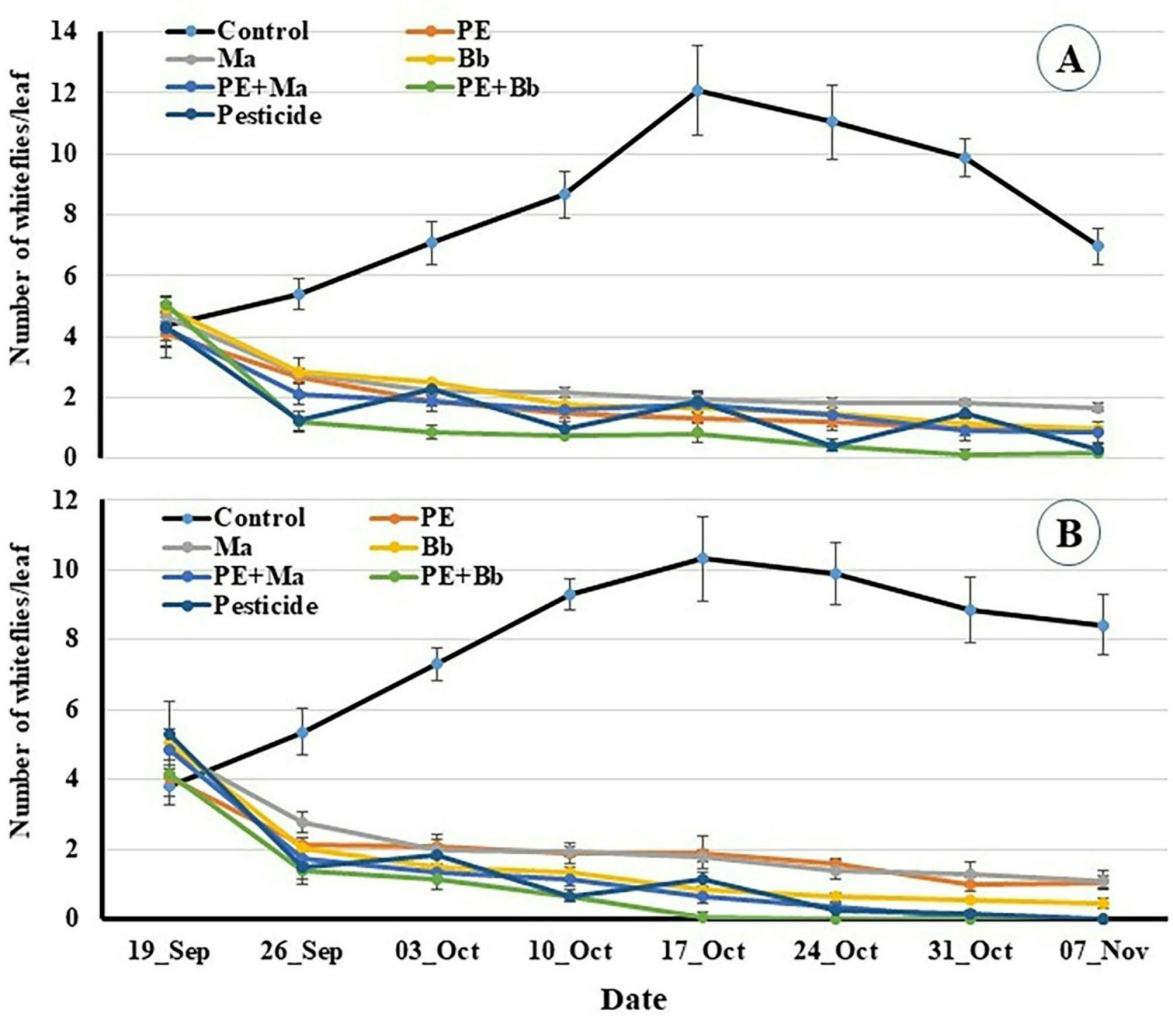
Population densities of whitefly, *Bemesia tabaci* on pepper plants treated with entomopathogenic fungi and *Calotropis procera* extract at location-1 (**A**) and location-2 (**B**).

The obtained results indicated that infestation rates of whitefy, *B. tabaci* at the beginning of the experiment on control plants were 4.37 and 3.83 whiteflies/leaf for location 1 and 2, respectively. These infestation’s rates gradually increased till the 4th week reaching 12.07 and 10.33 whiteflies/leaf, respectively. As in the aphid’s infestation rate, they gradually decreased till end of the experiment (6.97 and 8.43 whiteflies/leaf on November 07 for location 1 and 2, respectively) (Fig. 4A and B). The infestation rate in all treatments gradually decreased till end of the experiment in location-1 as follow: 0.93, 1.67, 0.97, 0.83, 0.17 and 0.27 whiteflies/ leaf ; while in location-2, they were 1.07, 1.1, 0.43, 0, 0 and 0 whiteflies/ leaf for PE, *Ma*, *Bb*, PE + *Ma*, PE + *Bb* and pesticide, respectively) (Fig. 4A and B).

**Fig. 5:**
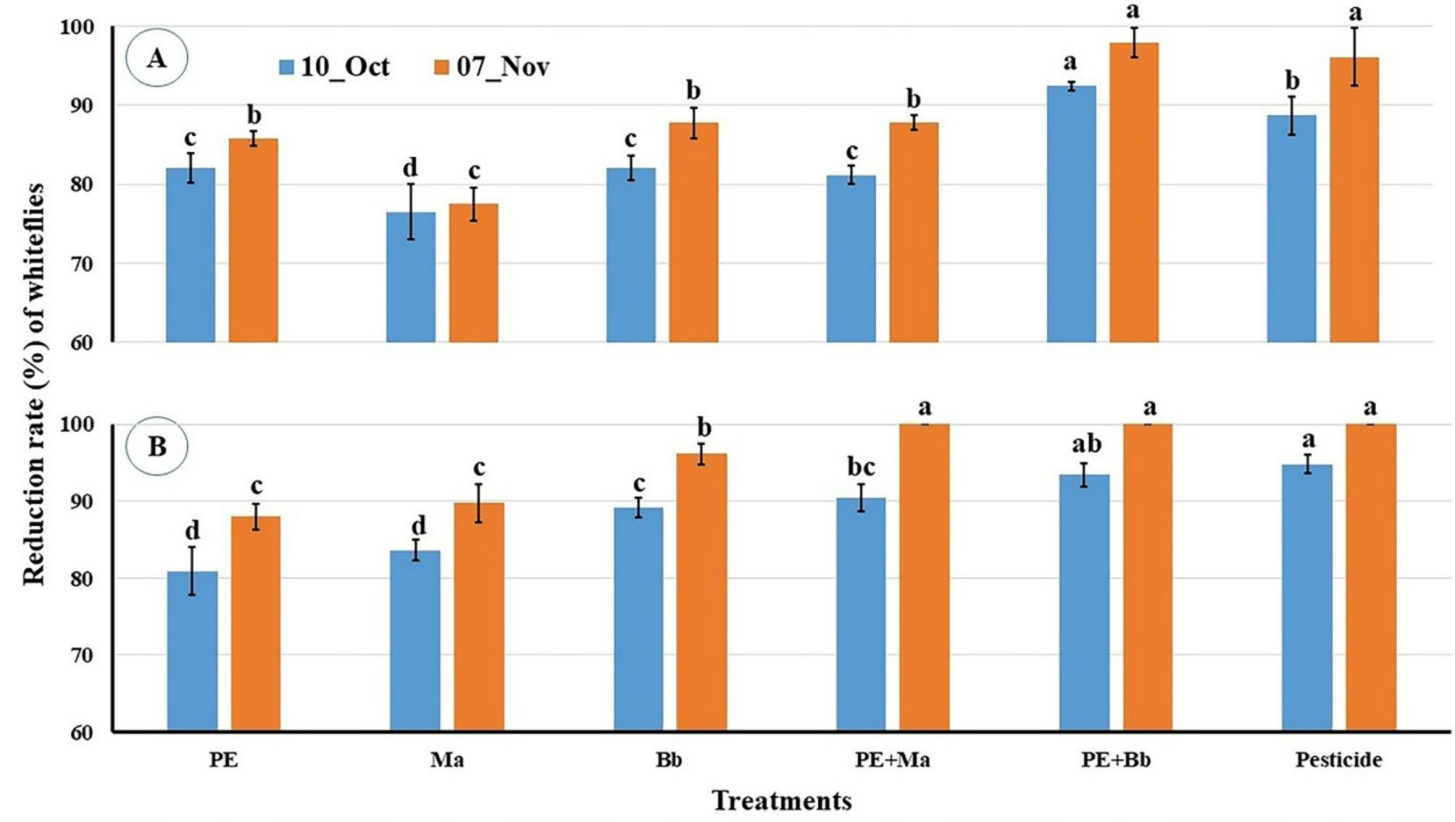
Reduction % in whitefly numbers post treatment with entomopathogenic fungi and *Calotropis procera* extract. **A** = location-1; **B** = location-2. Columns of the same period bearing different letters are significantly different with Duncan test (*α* = 0.05).

Whitefly’s reduction rates after three weeks of treatments showed significat differences among them in location-1 (F_5,12_ = 23.2, P < 0.001) where PE + *Bb* (92.39%) was significantly higher that that of pesticide (88.71%) follwed by *Bb* (81.98%), PE + *Ma* (81.16%) and PE (82.02%) and *Ma* (76.46.87%) (Fig. 5A). The reduction rates increased till end of experiments with significant differences among the treatments (F_5,12_ = 37.295, P˂0.001) by more than 96% for PE + *Bb* and pesticide followed by *Bb*, PE and PE + *Bb* (86–88%), and the lower rate of 77.48% (*Ma*). In the location-2, the same trend was achieved as in locaton-1 and also in the aphid infestation where the reduction rates after 3 weeks of treatments indicated significat differences among them (F_5,12_ = 27.12, P˂0.001). Meanwhile, PE + *Bb* (93.4%) and pesticide (94.82%) did not significantly different followed by PE + *Ma* (90.41%) and *Bb* (89.16%). The lower rates were 83.65% (*Ma*) and 80.92% (PE) (Fig. 5B). The end reduction rates reached 100% for the treatments of PE + *Bb*, PE + *Ma* and pesticide with significant differences (F_5,12_ = 49.84, P˂0.001) with *Bb* (96.11%) followed by the lower two treatments (PE = 87.98% and *Ma* = 89.76%) without a significant difference between both (Fig. 5B).

## Discussion

The plants even though belong to the same family or the same genus, their extracts contain various flavonoids and phenols and they have also various total contents of these compounds^18,21^. In our study, the PE of *C. procera* had reduction rates of about 80 and 87% for aphid and whitefly, respectively. Our investigation stated that there were 14 compounds of phenols and flavonoids. These compounds act as deterrents or as toxins for insect pests^22^. Flavonoids affecting the growth, behavior and development of insects and the mortality may be due to contact toxicity or the physiological changes in the body of insects^23^. Our investigation recorded a LC_50_ value of *C. procera* leaf extract on *M. persicae* of 292.24 µg/ml. This value is variable according to the component of the extract and the location of the collection. In this regard, the extract of *C. procera* leaves against *Musca domestica* (Muscidae: Diptera) showed a LC_50_ value of 282.5 µg/mL^24^.

The commercial product of *B. bassiana* recorded higher reduction of aphids and whiteflies in the current investigation than that of *M. anisopliae.* Many studies stated that there are differences in the infectivity of different species or isolates of EPF against the same insect pest^25,26^. Many investagations stated that the pathogenicity of EPFs is the isolate regardless of the fungus species. For examples; The EPFs significantly reduced the leaf damage of *Awash* tomato variety infested with the insect pest, *Tuta absoluta* Meyrick 1917 (Lepidoptera: Gelechiidae) where *B. bassiana*-AAUB03, *B. bassiana*-AAUB28, *M. anisopliae*-AAUM39 and *M. anisopliae*-AAUM78 achieved protection rates on leaves of 93.4, 86.1, 79.2 and 89.7%, respectively, while these rates on fruits were 93.5, 70.6, 76.9 and 94.4%, respectively^26^. The larval mortalities of *Spodoptera frugiperda* (Lepidoptera: Noctuidae) treated with five different isolates of *B. bassiana* were higher than that with an isolate of *M. anisopliae.* Moreover, the pathogenicities of the five isolates of *B. bassiana* were significantly different among them^27^. The pathogenicity of *B. bassiana* against four aphid species i.e., *Rhopalosiphum padi*,* Schizaphis graminum*,* Lipaphis erysimi* and *Brevicoryne brassicae* was higher than that of *M. anisopliae*^25^. With the concentration of 10^7^ spores/ml, the mortality rate was higher with *M. anisopliae* (83.3%) than that of *B. bassiana* (61.5%) against the cowpea aphid, *Aphis craccivora* Koch (Hemiptera: Aphididae)^28^. Also, the application of *B. bassiana* in this study recorded a higher reduction of both aphids and whiteflies than that of the plant extract. The same finding was obtained in different investigations with *B. bassiana* that produced significantly higher mortality on whitefly compared to plant extracts^29^.

In the present study, due to the probability of negative effect of the plant extract on the viability of EPF if they were combined together, the spray of the plant extract was done in the morning while the spray of EPF was done in the evening on the same day. Some investigations recorded negative effects for plant extracts on EPF such as the compatibility between *Annona squomosa* ethanol extract with EPF that recorded high reduction in the growth of all tested EPF, i.e.; *Lecanicillium lecanii* and *Isaria fumosorosea*^30^. A reduction of 45% in spore germination of *B. bassiana* was recorded when mixed with neem oil at 2%^31^.

The application of plant extracts from chinaberry neem, lantana and sunflower leaves reduced the growth of *B. bassiana*. Meanwhile, the combination of 0.25% chinaberry extract with *B. bassiana* (1 × 10^8^ conidia/ml) was most combatable with the conidial viability of 75.6% and might be used as promising natural alternatives to mycoinsecticides against *Spodoptera litura*^32^.

Synergism between plant extracts and EPF can enhance the infectivity and virulence of EPF. This maybe due to the bioactive compounds in plant extracts (such as phenols, flavonoids alkaloids, and terpenoids) making the insect host more susceptible to fungal infections by weakening its immune defenses, altering its behavior, or disrupting its cuticle, making it easier for the fungal spores to penetrate the insect’s body cuticle. This can increase the likelihood of successful infection and faster fungal growth^33^.

Some plant extracts can stimulate fungal spore germination. The chemicals in the extracts may promote the development of the fungal hyphae, leading to quicker colonization and infection of the pest, such as essential oils (e.g., neem oil or clove oil) that can stimulate fungal growth and help in the formation of conidia; and thus increases the efficacy of biological control^34^.

In general, using of plant extracts and EPF in IPM programs offers a broad-spectrum control strategy where plant extracts may target specific pests such as aphids, whiteflies or thrips, the EPF may infect a various range of insect pests, providing a more comprehensive pest management solution^35,36^.

The present study showed that the application of both *Bb* and PE without combination had the higher reductions in numbers of both aphids and whiteflies (96 to 100%) followed by *Bb* and PE without combination. Both treatments were significantly higher than those obtained with PE, *Bb* or *Ma* individually. There are successful synergistic effects and compatibilities between EPF and various plant-derived pesticides to improve the insect pest control. In this regard, Islam and Omar^37^ indicated that the combination of *B. bassiana* (10^8^ conidia/ml) with neem extract is compatible and achieved the higher mortality of *B. tabaci* in eggplant. A combination of *M. anisopliae* and pyrethrum achieved the higher rate of mortality in *Aphis fabae* than the insecticide without combination^14^. Ali et al. indicated that EPF and botanical extracts of eucalyptus or neem caused a significant reduction in survival and fecundity of Aphids^38^. The leaf extract of neem is not toxic to the conidia of *B. bassiana* or they are compatible when combined in the application^39^. Other investigation stated that four plant extracts (*Salvia officinalis*,* Pulicaria crispa*, *Euryops arabicus*, and *Ochradenus baccatus*) were compatible with *B. bassiana* against *Aphis gossypii* while *Psiadia penninervia* extract was not compatible with *B. Bassiana*^40^. EPFs include *B. bassiana* are thought to be safe for pollinators but little is known about its side effects on pollinators’ cognition and behavior. Moreover, the bees exposed to EPFs were generally less responsive to odorants than the control bees. Therefore, it is important to assess the effect of any used EPF isolate on honey bee to make sure from its safety on pollinators and other beneficial insects such as natural enemies^41^. Nonetheless, to present, even though EPF have been commercialized in the last decades, their broad potential applications have not yet been fully discovered^42^. The application of EPF through inoculation of some plant parts rather by means of inundation which is contingent upon exposing spores to unfavorable environmental conditions, could minimize the risks of abiotic factors^43^. However, to present, the EPF products remain underutilized due to high cost of formulations, the high humidity needed during their application, the short shelf life of the inoculum, and the long period required until mortality occurs. Meanwhile, in the IPM programs, biological control of pests has become a more than necessary control approach as chemical pesticides overburdens the environment and products with harmful residues, and consumers are conscious of the safety of their products^44^. The application of combined EPFs and plant extracts is important but we have to make sure from the compatibility between them due to some combination are not successive. Compatibility between *B. bassiana* at various concentrations with plant leaf extracts of Chinaberry, Neem, Mexican sunflower, and Lantana against larvae of *Spodoptera litura* (Lepidoptera: Noctuidae) has been investigated to determine conidial viability, colony growth, and insect mortality. Results indicated that the colony growth decreased in all combinations while the most suitable combination was of 0.25% fungus concentration of Chinaberry while *B. bassiana* achieved the higher conidial density and viability, and insect mortality (44%)^32^. The compatibility of *B. bassiana* with emulsible neem oil, *Azadirachta indica* and with neem seeds and aqueous extracts of leaves indicated that the latter did not affect the conidial viability of the fungus although production and conidia growth were reduced. In contrast, the emulsible neem oil at higher concentration, affected all conidia parameters, thus leading to non-compatible with the fungus^45^. Accordingly, in the current investigation, to prevent the side effect of the plant extract, the plant extract was sprayed in the morning while the fungus was sprayed in the evening. Long-term application of EPFs gives a great chance for fungal colonization in the plant to be endophytic EPFs. Thus, foliar application coupled with endophytic colonization provide a dual role of enhancing plant protection as well as overcoming the hurdles of conidial exposure to non-suitable environmental conditions that foliar application entails^29^.

## Conclusion

The application of both *B. bassiana* and *M. anisopliae* with *C. procera* extract without combinations achieved the higher reduction rates against aphids and whiteflies (96 to 100%) and were significantly higher than the reduction rates of the single applications of each. Therefore, using of both EPFs and *C. procera* extract without combination as biopesticides in the sustainable agriculture is an environmentally safe approach to control insect pests. Moreover, this plant extract could be evaluated against the important natural enemies to state its safety for them.

## Data Availability

The datasets used and/or analysed during the current study available from the corresponding author on reasonable request.
